# Faecal and Urinary Incontinence after Multimodality Treatment of Rectal Cancer

**DOI:** 10.1371/journal.pmed.0050202

**Published:** 2008-10-07

**Authors:** Marilyne M Lange, Cornelis J. H van de Velde

## Abstract

Marilyne Lange and Cornelis van de Velde discuss the differential diagnosis and management of incontinence after rectal cancer treatment.

## DESCRIPTION of CASE

During a control check-up after rectal cancer treatment, a 67-year-old woman reported experiencing frequent bowel movements. Five years before, she was treated for a stage I tumour (T2N0M0), located 5 cm from the anal verge. The treatment consisted of short-course pelvic radiotherapy (5 × 5 Gy), followed by low anterior resection (LAR) with straight colorectal anastomosis. No post-operative complications had occurred, and a temporary colostomy had been constructed, which was reversed after four months. The woman had no previous medical history.

On questioning, she reported having suffered from rectal urgency symptoms ever since the colostomy was reversed. These symptoms were quite acceptable, especially because she assumed them to be temporary. However, they became worse over time, and urgency developed into involuntary stool leakage, requiring her to wear a protective pad constantly. Furthermore, she indicated that she experienced urine loss, especially during coughing, laughing, or lifting. She stated that voiding and defecation were fully normal before her treatment for rectal cancer.

### Are Incontinence Problems after Rectal Cancer Treatment Common?

**Figure 1:**
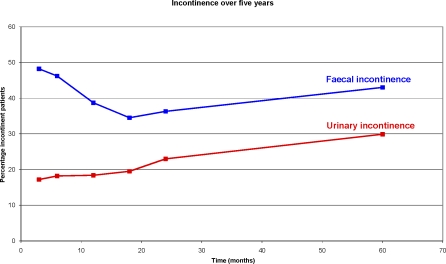
Percentage of Faecal and Urinary Incontinence after Rectal Cancer Treatment Reported by Patients without Pre-Operative Faecal or Urinary Incontinence Who Participated in the Dutch TME Trial [16]

Faecal incontinence after rectal cancer treatment occurs frequently, affecting almost half of patients with normal pre-operative functioning [1]. Incontinence may range from inadvertent gas release to minor soiling or complete escape of rectal contents. These symptoms are often described as “anterior resection syndrome” [2]. Long-term urinary incontinence develops in almost one third of patients, and combined urinary and faecal incontinence occurs in 14% of patients with normal pre-operative function [3,4]. Figure 1 shows the incidence of faecal and urinary incontinence five years after rectal cancer treatment. Incontinence problems lead to avoidance of certain activities, such as long-distance travel by car or plane, during which bathroom facilities may not be immediately available. Additionally, sexual dysfunction is a frequent and distressing complication of rectal cancer treatment. Male patients may experience ejaculatory problems and impotence. Female patients may suffer from dyspareunia and vaginal dryness [5]. The poor functional outcome of rectal cancer treatment is a major problem, since bowel and urinary dysfunction can have a negative impact on a patient's physical, psychological, social, and emotional functioning, as well as the patient's overall well-being [6].

### What Could Have Caused Her Incontinence Problems?

Faecal incontinence is usually the result of failure of more than one component of the continence mechanism. The rectum, the anal sphincters, and the pelvic floor muscles are essential in the maintenance of faecal continence [7]. First of all, the rectum acts as a reservoir to store stool. A smaller neorectum after LAR has a lower capacity, causing a decrease in maximum tolerated volumes. Furthermore, the anal canal contains a rich network of nerve endings sensitive to pain, temperature, and touch, which is used to differentiate solid or liquid stool from flatus, and allows for selective passage of flatus. Patients with a small neorectum after resection of a low-lying tumour, as was the case in the present patient, are therefore at increased risk for faecal incontinence. Moreover, in this patient's case, rectal cancer resection was preceded by pelvic radiotherapy, which is known to increase the risk of faecal incontinence [8]. Radiotherapy diminishes compliance of the rectum due to fibrosis, resulting in a reduced reservoir function. Radiotherapy-induced fibrosis of the myenteric plexus of the internal anal sphincter can prevent adequate closure of the anal canal in a resting state. In addition, faecal incontinence after rectal cancer treatment has been reported to be caused by impaired pelvic floor movement, i.e., a disturbed change in anorectal angle due to a dysfunctional levator ani muscle [9]. According to the latest anatomical insight, the levator ani muscle is not innervated by the pudendal nerve, but receives its innervation from a separate nerve, which runs on the surface of the pelvic floor (a three-dimensional reconstruction of the levator ani nerve can be obtained online in a recent publication in the *Journal of Clinical Oncology* [4]). During surgical dissection deep within the pelvis, especially in the case of a low-lying tumour, this nerve might be disrupted [4].

**Figure 2:**
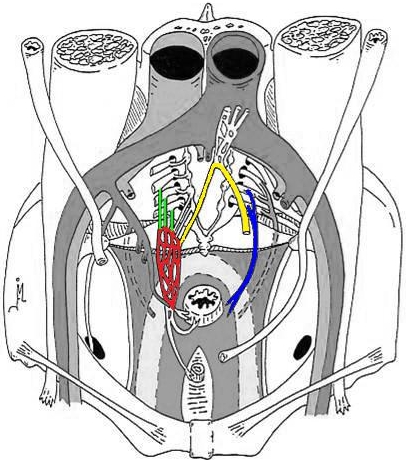
Anatomical Graph of the Pelvic Floor and the Autonomic Nerves Yellow: hypogastric nerves; green: pelvic splanchnic nerves; red: pelvic plexus; blue: levator ani nerve. Adapted from: Lange JF (2002) Surgical anatomy of the abdomen. Maarssen (Netherlands): Elsevier. p. 178.

Urinary incontinence after rectal cancer treatment may consist of urge, overflow, and/or stress incontinence. Urge incontinence may result from a reduced bladder capacity due to surgical disruption of the sympathetic nerve supply (the hypogastric nerves and the pelvic plexus; Figure 2) [10]. Overflow incontinence may be caused by surgical damage to the sacral splanchnic nerves, resulting in bladder emptying problems (Figure 2). However, the present patient suffered from involuntary urine loss during increased abdominal pressure, which is a sign of urinary stress incontinence. Stress urinary incontinence may result from impaired support to the urethra and bladder neck. This support is regulated by surrounding structures, the most important being the pubourethral-vesical ligaments, the suburethral vaginal wall, the levator plate, the pubococcygeus muscles, and the connective tissue. These components can compensate for each other in case of inappropriate function. In post-menopausal female patients, many of these structures are impaired because their function is influenced by oestrogen receptors. Additional changed anatomical relations between bladder, urethra, and pelvic floor, and possibly damage to the innervation of the levator ani muscles during LAR, would further impair the continence mechanism and lead to urinary incontinence [11].

### What Could Have Caused the Worsening of Symptoms Over Time?

**Figure 3:**
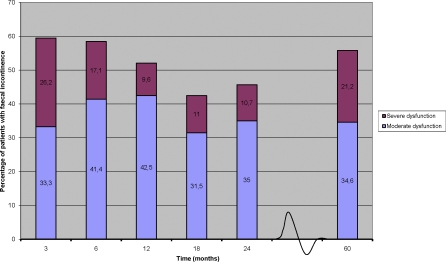
Incidence of Moderate and Severe Faecal Incontinence Reported by Patients Who Participated in the Dutch TME Trial [16] and Were Treated with Pre-Operative Radiotherapy Only patients without pre-operative dysfunction were included in this diagram.

Improvement in anorectal function usually occurs over six to 12 months, which correlates with expansion in the reservoir capacity of the neorectum. Post-operative assessment of function after one year has shown that many patients achieve continence to solid stool, but that control of minor staining, flatus, and stool frequency is more variable [12]. However, worsening of faecal incontinence over time is typically seen in patients treated with pelvic radiotherapy (Figure 3). This may be explained by the fact that vascular damage is a long-term effect of radiotherapy, compromising the endovascular cushions filling the 7–8 mm gap within the internal sphincter ring, which contribute to continence at rest [13]. Worsening of urinary incontinence over time could be due to ageing and laxity of the pelvic floor musculature, with concomitant risk of increased stress incontinence.

### How Could Incontinence Problems Have Been Prevented?

First, functional problems after rectal cancer surgery might have been prevented by special attention to nerve preservation. Despite the fact that the current rectal resection technique, described by total mesorectal excision (TME), permits preservation of the innervation of the pelvic organs, nerve-sparing surgery can be difficult. Differences among individuals in the running patterns of the nerves and variation in the volumes of nerve fibres in each region of the pelvis hamper appropriate identification of structures. Especially during excessive peroperative blood loss, nerves are at risk due to diathermic coagulation and numerous sutures to secure haemostasis. To avoid excessive bleeding and accidental nerve disruption during surgery, it is important to adhere to the surgical plane and reduce the use of blunt dissection. Also, the use of a nerve-stimulating device may facilitate preservation of the pelvic autonomic nerves during pelvic dissection [14].

It is difficult to prevent radiotherapy-induced bowel dysfunction, as radiotherapy is considered an important part of rectal cancer treatment, decreasing the risk of local recurrence [15,16]. However, treating all rectal cancer patients with radiotherapy can result in substantial overtreatment. Therefore, it is of great importance to identify patients with low risk of local recurrence for whom radiotherapy is redundant [16]. Currently, new imaging modalities are being developed and molecular biomarkers are being identified to predict prognosis, making patient-tailored treatment a possibility soon.

An alternative to avoid faecal incontinence after rectal cancer treatment is to construct a permanent stoma by abdominoperineal resection instead of sphincter preservation by LAR. Traditionally, the construction of a stoma has been regarded as an unfavourable outcome, as the quality of life (QOL) experienced by stoma patients is believed to be inferior compared to non-stoma patients. However, recent studies have shown that QOL after abdominoperineal resection may not be as bad as once believed and may be equal to that after LAR [17]. Moreover, LAR patients with a low anastomosis have been reported to have a worse QOL than stoma patients, due to poor functional outcome [18]. Obviously, cultural, social, religious, and sociodemographic factors influence how patients assess their QOL with a permanent stoma. In selected cases, abdominoperineal resection might be a more satisfactory option than LAR.

### What Treatment Modalities Are Available?

Standards of management of patients with incontinence problems after rectal cancer treatment are still lacking. Many treatments are available, but there is not enough evidence to support the effectiveness of any of them. First of all, conservative measures aimed at symptomatic control (e.g., dietary regiments, absorbent pads, and pharmacotherapy including hormonal manipulation, constipating agents, and enemas) may be tried. Colonic irrigation in the morning in order to clean the colon of faeces has been shown to reduce symptoms [19]. The present patient was offered colonic irrigation. During a control check-up one year later, she reported a beneficial effect of colonic irrigation, improving the quality of her life.

Alternatively, there are a number of interventions aimed at correcting the underlying cause, including both non-surgical and surgical techniques. Non-surgical procedures include biofeedback and pelvic floor muscle training. Biofeedback therapy, showing patients how to use the pelvic floor muscles properly, is often recommended and may consist of rectal sensitivity, strength, and coordination training [20]. Pelvic floor muscle training improves pelvic floor support. It is regarded as the first-line treatment for urinary incontinence and used to improve faecal incontinence. Pelvic floor muscle training may be of limited use in patients in which the innervation of the pelvic floor has been damaged during surgery.

If conservative management fails, surgical intervention may be considered. First, appropriate assessment should be carried out for characterisation of the underlying cause. Anorectal physiology can be evaluated with manometry, and mechanical damage to the sphincter muscle can be detected with endoanal ultrasound. If the sphincter is intact, sacral nerve stimulation, in which electrodes are inserted through the sacral foramina under general anaesthetic for stimulation of the sacral nerves, may be effective for both urinary and faecal incontinence. Several studies have shown promising results; however, experience with sacral nerve stimulation for faecal incontinence following rectal resection is still limited [21]. In case of a sphincter muscle defect, an artificial bowel sphincter can be constructed. This would not be an option in the present case, as severe complications after artificial bowel sphincter construction in a radiation-injured anorectum have been reported [22]. The construction of a colostomy is considered an option when all else has failed, but is also associated with a significant rate of complications in irradiated patients [23]. Uncertainty remains whether any surgical intervention does more good than harm.

## DISCUSSION

Incontinence problems after rectal cancer treatment are common and can have a major impact on QOL. Nevertheless, this issue often remains undiscussed in clinical practice. Patients should be informed pre-operatively about the possible development of anorectal and urogenital dysfunction and the increased risk in case of a low-lying tumour and radiotherapy. The different surgical options, LAR and abdominoperineal resection, and their potential outcomes should be discussed with the patient, as the individual preference of the patient is of great importance in this situation [24]. Education on modern stoma care may reduce ill-informed hesitations towards a permanent stoma by patients. At present, there is an ongoing project concerning the perceived costs and benefits of pre-operative radiotherapy in rectal cancer treatment, which is investigating to what extent patients and oncologists believe that patients should also participate in decision-making regarding pre-operative radiotherapy.

Post-operatively, evaluation of the patient's functional outcome should be standard procedure at every follow-up appointment. Patients are not likely to bring up incontinence problems themselves, out of shame or because they don't relate it to rectal cancer treatment, especially if incontinence problems occur several years after treatment. Available therapies, primarily conservative regiments, should be proposed if needed.

Although the exact aetiology of incontinence problems after multimodality treatment of rectal cancer is unknown, specific technical aspects of the surgical procedure play a major role. Special attention to pelvic autonomic nerves, sharp dissection, adhering to the surgical plane, and reducing the use of blunt dissection may lower the risk of urinary and faecal incontinence [4]. Furthermore, to prevent overtreatment with radiotherapy, a tailor-made approach for every rectal cancer patient that is based on pre-operative prediction of the risk for local recurrence seems to be favourable. Currently, new imaging modalities are being developed, and molecular biomarkers have been identified that predict prognosis, making patient-tailored treatment possible soon [25,26]. This will reduce the number of patients with poor functional outcome after rectal cancer treatment.

**Key Learning Points**

- Incontinence problems after rectal cancer treatment are common and can have a major impact on QOL.
- Patients should be informed about the surgical options, LAR and abdominoperineal resection, and about their potential outcomes.
- Surgical nerve damage may play a major role in the development of faecal and urinary incontinence, with an additional effect of radiotherapy.
- Faecal incontinence can worsen over time in case of radiotherapy.
- Several non-surgical and surgical therapies for incontinence problems are available; conservative therapies should be the first line-choice.

## Abbreviations

LAR: low anterior resection
QOL: quality of life
TME: total mesorectal excision
